# Supplementary material for: Exploring the facilitators, barriers, and strategies for self-management in adults living with severe mental illness, with and without long-term conditions: A qualitative evidence synthesis
Source: PLoS One. 2021 Oct 26;16(10):e0258937. doi: 10.1371/journal.pone.0258937 (PMC8547651; doi:10.1371/journal.pone.0258937)
Supplement: S1 Appendix. Revisions to systematic review protocol — (DOCX) [file pone.0258937.s002.docx]

**Appendix 1. Revisions to systematic review protocol**

| **Section** | **Original protocol** | **Revised protocol** | **Rationale** |
| --- | --- | --- | --- |
| Inclusion criteria | Inclusion of quantitative study designs | Inclusion of qualitative research only | Studies were screened and then grouped by quantitative/qualitative design. The quantitative and qualitative studies are reported separately. |
| Inclusion criteria | No restrictions on setting | Exclusion of studies of inpatients | Inpatients are usually acutely unwell with a relapse of SMI, so self-management is likely to differ during this period. The inpatient setting is likely to involve different experiences of self-management |
| Inclusion criteria | Inclusion of only adults (age 18 and over) with severe mental illness (with and without long-term physical conditions) | Inclusion of people providing care and support to adults with severe mental illness (with and without long-term physical conditions). Including healthcare professionals and inform carers . | People who provide support and care may be able to provide insight on the barriers, facilitators and strategies towards self-management that are experienced by the people they support with severe mental illness. |
| Quality appraisal | Assessment of study quality using a framework developed for mixed methods reviews | Use of the data richness scale developed by Ames et al. (2017 ) to score each study according to data richness (as a measure of quality) and relevance (to the review aim | Use of a tool more appropriate for the design of qualitative studies |
| Sampling | Studies included if they meet the eligibility criteria | Purposively sampled qualitative data, according to data richness scores. | Large number of studies and ensure studies with the most appropriate and rich data contributed to data saturation. |
| Data synthesis | Narrative synthesis of determinants of self-management  using the Capabilities, Opportunities, Motivations and Behaviours (COM-B) system | Inductive thematic synthesis. | Use of a form of synthesis appropriate for qualitative evidence synthesis. COM-B framework did not adequately capture data. |
