# Supplementary material for: Exploring the facilitators, barriers, and strategies for self-management in adults living with severe mental illness, with and without long-term conditions: A qualitative evidence synthesis
Source: PLoS One. 2021 Oct 26;16(10):e0258937. doi: 10.1371/journal.pone.0258937 (PMC8547651; doi:10.1371/journal.pone.0258937)
Supplement: S2 Appendix — (DOCX) [file pone.0258937.s003.docx]

**Appendix 2 Ovid Medline Search Strategy**

| Database: Ovid MEDLINE(R) <1946 to July Week 2 2018> |
| --- |
| 1 (bipolar adj (disorder* or disease* or illness*)).tw,kf. (21737)  2 exp schizophrenia/ (97897)  3 Affective disorders, psychotic/ (2204)  4 Bipolar disorder/ (37267)  5 paranoid disorders/ (3973)  6 exp psychotic disorders/ (48180)  7 schizo*.tw,kf. (119094)  8 (mani* adj3 depress*).tw,kf. (8267)  9 (psychotic* adj3 depress*).tw,kf. (2369)  10 (severe* adj3 affective*).tw,kf. (207)  11 (severe* adj3 mental*).tw,kf. (9026)  12 (severe* adj3 depress*).tw,kf. (8801)  13 (psychos#s adj3 depress*).tw,kf. (3368)  14 (serious* adj3 affective*).tw,kf. (39)  15 "serious mood*".tw,kf. (24)  16 (serious* adj3 mental*).tw,kf. (3957)  17 (serious* adj3 depress*).tw,kf. (616)  18 or/1-17 [Serious Mental Illness] (223362)  19 self care/ (30488)  20 self administration/ (10593)  21 Self Medication/ (4458)  22 Self Efficacy/ (17300)  23 Self-Management/ (556)  24 Self help groups/ (8641)  25 blood glucose self-monitoring/ (5606)  26 (self adj2 (efficac* or help or care* or cure* or manage* or directed or monitor* or medicat* or treat* or inject* or remed*)).tw,kf. (65658)  27 (selfefficac* or selfhelp or selfcare or selfcure* or selfmanage* or selfdirected or selfmonitor* or selfmedicat* or selftreat* or selfinject* or selfremed*).tw,kf. (185)  28 (self administ* not (self administ* adj2 (interview? or survey? or questionnaire?))).tw,kf. (15986)  29 or/19-28 [Self Management only terms] (119143)  30 life style/ or exp healthy lifestyle/ or life change events/ or sedentary lifestyle/ (81876)  31 exp Diet Therapy/ or exp Diet/ or exp Food/ or exp Feeding Behavior/ (1407344)  32 exp Exercise/ (167064)  33 smoking cessation/ or smoking reduction/ (25689)  34 Alcohol Abstinence/ (457)  35 *health promotion/ or *healthy people programs/ or *weight reduction programs/ (45563)  36 ((behavio?r or lifestyle or "life style" or habit?) adj2 (chang* or improv* or modif*)).tw,kf. (41849)  37 ((diet* or eating) adj2 (healthy or improv*)).tw,kf. (13706)  38 (physical adj1 (activit* or exercise*)).tw,kf. (89158)  39 ((Smoking or cigar* or tobacco or alcohol*) adj2 (cessation or stop* or quit* or reduc* or abstinen* or withdrawal*)).tw,kf. (42214)  40 (weight adj (loss or reduction)).tw,kf. (70109)  41 exp "treatment adherence and compliance"/ (215866)  42 ((adher* or non-adher* or compliance or non-compliance) adj2 (treatment? or medication*)).tw,kf. (21050)  43 (screening adj5 (health* or cancer*)).tw,kf. (47461)  44 or/30-43 [Healthy lifestyle] (2041439)  45 *Patient Education as Topic/ (36279)  46 exp Social Support/ (64039)  47 (social adj2 support*).tw,kf. (30139)  48 Patient care planning/ (37203)  49 or/45-48 [Patient Knowledge] (147247)  50 barrier?.ti,kf. (44604)  51 difficult*.ti,kf. (26242)  52 weakness*.ti,kf. (3988)  53 participat*.ti,kf. (31623)  54 facilitat*.ti,kf. (31063)  55 enabler*.ti,kf. (365)  56 strength*.ti,kw. (37766)  57 determinant*.ti,kf. (44930)  58 (("Theoretical Domain?" or "Implementation Research" or Ecological or "Knowledge to Action" or "COMB B") adj4 (Framework* or model? or system?)).tw,kf. (6077)  59 motivat*.ti,kf. (16867)  60 promot*.ti,kf. (142138)  61 goal?.ti,kf. (15048)  62 uptake.ti,kf. (62745)  63 problem?.ti,kf. (179071)  64 ((tackl* or address* or solv* or resolv* or sort*) adj1 problem*).tw,kf. (18324)  65 Problem Solving/ (23501)  66 exp Motivation/ (155294)  67 or/50-66 [Barriers or Motivators] (783038)  68 or/44,49 [Healthy lifestyle or patient knowledge] (2157107)  69 67 and 68 [Barriers to healthy lifestyle or knowledge] (125499)  70 (barriers adj4 care).ti. (1230)  71 29 or 69 or 70 [Self Management or Barriers to lifestyle change] (237043)  72 18 and 71 [SMI and SM or barriers to lifestyle change] (3836)  73 Comment/ (678448)  74 letter/ (937414)  75 editorial/ (411497)  76 note/ (1988)  77 news/ (174511)  78 newspaper article/ (18274)  79 (comment* or letter? or editorial? or note?).ti. (163738)  80 case reports/ (1881218)  81 or/73-80 (3456232)  82 Published Erratum/ or Retraction of Publication/ (5530)  83 81 not 82 [Comments/Letters] (3455452)  84 72 not 83 [SMI and SM or barriers to lifestyle change editorials etc... removed] (3531)  85 exp Animals/ not exp Humans/ (4473346)  86 (adolescent/ or child/ or infant/) not exp adults/ (1425928)  87 84 not (85 or 86) [SMI and SM or barriers to lifestyle change - editorials/children/animals removed] (3280) |
