# Supplementary material for: Exploring the facilitators, barriers, and strategies for self-management in adults living with severe mental illness, with and without long-term conditions: A qualitative evidence synthesis
Source: PLoS One. 2021 Oct 26;16(10):e0258937. doi: 10.1371/journal.pone.0258937 (PMC8547651; doi:10.1371/journal.pone.0258937)
Supplement: S3 Appendix — (DOCX) [file pone.0258937.s004.docx]

**Appendix 3. Data richness scale used for quality appraisal (Ames et al., 2017) [29]**

| **Score** | **Criteria** | **Example** |
| --- | --- | --- |
| 1 | Very few qualitative data presented that relate to the synthesis objective. Those findings that are presented are fairly descriptive. | For example, a mixed methods study using open ended survey questions or a more detailed qualitative study where only part of the data related to the synthesis objective. |
| 2 | Some qualitative data presented that relate to the synthesis objective. | For example, a limited number of qualitative findings from a mixed methods or qualitative study. |
| 3 | A reasonable amount of qualitative data that relate to the synthesis objective. | For example, a typical qualitative research article in a journal with a smaller word limit and often using simple thematic analysis. |
| 4 | A good amount and depth of qualitative data that relate to the synthesis objective. | For example, a qualitative research article in a journal with a larger word count that includes more context and setting descriptors and a more in-depth presentation of the findings. |
| 5 | A large amount and depth of qualitative data that relate in depth to the synthesis objective. | For example, from a detailed ethnography or a published qualitative article with the same objectives as the synthesis. |
