# Supplementary material for: Exploring the facilitators, barriers, and strategies for self-management in adults living with severe mental illness, with and without long-term conditions: A qualitative evidence synthesis
Source: PLoS One. 2021 Oct 26;16(10):e0258937. doi: 10.1371/journal.pone.0258937 (PMC8547651; doi:10.1371/journal.pone.0258937)
Supplement: S4 Appendix — (DOCX) [file pone.0258937.s005.docx]

**Appendix 4. Summary of studies eligible for review.**

| First author, year  Country | SMI of interest | LTC of interest | Theoretical approach/analysis method(s)  Data collection method | DRS | Included in final synthesis Y/N |
| --- | --- | --- | --- | --- | --- |
| SMI and LTC | | | | | |
| Cimo, 2018 [1]  Canada | Schizophrenia  BD  Depressive disorder  Severe anxiety  Multiple diagnoses | DM/pre-DM  ‘borderline DM’  ‘borderline high sugars’  ‘slightly high blood sugar levels’ | Thematic analysis  Focus groups | 5 | Y |
| Knyahnytska, 2018 [2]  Canada | Schizophrenia  BD | T2DM | Critical ethnography  Interviews | 5 | Y |
| Mulligan, 2017 [3]  UK | Schizophrenia  Schizoaffective disorder  BD  Personality disorder  Depression with psychotic features | T2DM | Analysis informed by theoretical domains framework  Interviews | 5 | Y |
| Blixen, 2016a [4]  USA | Schizophrenia or schizoaffective disorder  BD  Major depression | DM (type not specified) | Phenomenology  Interviews | 5 | Y |
| El-Mallakh, 2006 [5]  USA | Schizophrenia  Schizoaffective disorder | T1DM  T2DM | Grounded theory/constant comparison method  Interviews | 5 | Y |
| Stenov, 2020 [6]  Denmark | Schizophrenia  BD  Personality disorder  Severe depression | T1DM  T2DM | Systematic text condensation  Interviews | 4 | Y |
| Lowndes, 2013 [7]  Canada | Schizophrenia  Schizoaffective disorder  BD  ‘other form of mental illness’ | DM (type not specified)  Pre-diabetes | Institutional ethnography  Interviews | 3 | N |
| Arbour-Nicitopoulos, 2011 [8]  Canada | BD  Schizophrenia  Anxiety disorders  Major depression | Osteoporosis  Arthritis  Emphysema  Bronchitis  COPD  Cancer | Standard thematic analysis/constant comparative method  Interviews | 3 | N |

| SMI only |
| --- |

| Jimenez, 2017 [9]  USA | Schizoaffective disorder  Schizophrenia  Severe major depressive disorder  BD | N/A | Thematic analysis  Interviews | 5 | Y |
| --- | --- | --- | --- | --- | --- |
| Chee, 2019 [10]  Australia | Psychosis | N/A | Grounded theory  Interviews | 4 | Y |
| Nakanishi, 2019 [11]  Japan | Schizophrenia | N/A | Content analysis  Panel discussions during workshop | 4 | Y |
| Blixen, 2018 [12]  USA | BD | N/A | Content analysis with an emphasis on dominant themes  Focus groups | 4 | Y |
| Wheeler, 2018 [13]  Australia | Schizophrenia  BD  Depression  Agoraphobia  Multiple diagnoses | N/A | Interpretive phenomenological analysis  Interviews | 4 | Y |
| Blixen, 2016b [14]  USA | BD Type1  BD Type 2 | N/A | Thematic analysis  Interviews | 4 | Y |
| Rastad, 2014 [15]  Sweden | Schizophrenia  Schizoaffective disorder | N/A | Conventional qualitative content analysis  Interviews | 4 | Y |
| Shor, 2013 [16]  Israel | SMI not specified | N/A | Grounded theory  Interviews | 4 | Y |
| Johnstone, 2009 [17]  UK | Schizophrenia | N/A | Interpretive phenomenological analysis  Interviews | 4 | Y |
| Keller-Hamilton, 2019 [18]  USA | Schizophrenia  Schizoaffective disorder  Bipolar disorder with psychotic features  Psychosis not otherwise specified | N/A | Thematic analysis  Focus groups | 3 | Y |
| Ehrlich, 2018 [19]  Australia | Schizophrenia  DB  Depression | N/A | Thematic analysis/constant comparative process  Interviews | 3 | N |
| Heffner, 2018 [20]  USA | BD type 1  BD type 2 | N/A | Inductive content analysis  Interviews | 3 | Y |
| Lundstrom, 2017 [21]  Sweden | Schizophrenia | N/A | Phenomenological hermeneutic approach  Interviews | 3 | N |
| Firth, 2016 [22]  UK | Non-organic psychosis  Schizophrenia  Schizoaffective disorder  BD  Other psychotic disorder | N/A | Thematic analysis  Interviews | 3 | N |
| Sundgren, 2016 [23]  Sweden | Schizophrenia | N/A | Hermeneutic phenomenological approach  Interviews | 3 | N |
| Kemp, 2015 [24]  Australia | SMI not specified | N/A | Naturalistic inquiry  Focus groups | 3 | N |
| Van den Heuvel, 2015 [25]  Netherlands | BD | N/A | Phenomenology  Interviews | 3 | N |
| Villaggi, 2015 [26]  Canada | Depression  Anxiety  BD | N/A | Thematic analysis  Interview | 3 | N |
| Leutwyler, 2014 [27]  USA | Schizophrenia  Schizoaffective disorder | N/A | Grounded theory/constant comparison analysis approach  Interviews  Focus groups | 3 | N |
| Pearsall, 2014 [28]  UK | Schizophrenia  Schizoaffective disorder  Bipolar affective disorder | N/A | Grounded theory/thematic analysis  Interviews | 3 | Y |
| Rӧnngren 2014 [29]  Sweden | SMI not specified | N/A | Manifest content analysis  Focus groups | 3 | N |
| Katakura, 2013 [30]  Japan | Schizophrenia | N/A | Inductive qualitative approach  Interviews | 3 | N |
| Leutwyler, 2013a [31]  USA | Schizophrenia  Schizoaffective disorder | N/A | Grounded theory/constant comparison analysis approach  Interviews | 3 | N |
| Wardig, 2013 [32]  Sweden | Schizophrenia  Schizoaffective disorder  BD  Delusional disorder  Unspecified psychosis | N/A | Conventional content analysis  Interviews | 3 | Y |
| Williams, 2013 [33]  Australia | Schizophrenia  Psychosis  BD | N/A | Thematic analysis  Interviews | 3 | Y |
| Barre, 2011 [34]  USA | Schizophrenia/schizoaffective disorder  BD  Major depression | N/A | Thematic analysis  Interviews | 3 | Y |
| Suto, 2010 [35]  Canada | BD type 1 and 2 | N/A | Thematic analysis  Interviews  Focus groups | 3 | N |
| Schmutte, 2009 [36]  USA | SMI not specified | N/A | Interpretive phenomenological analysis  Focus groups | 3 | N |
| Carless, 2007 [37]  UK | Schizophrenia  Schizophrenic illness | NA | Ethnography/content analysis/mental map  Interviews | 3 | N |
| Soundy, 2007 [38]  UK | Schizophrenia  BD  Manic depression  Social anxiety | N/A | Thematic analysis  Interviews | 3 | N |
| McDevitt, 2006 [39]  USA | Schizophrenia or non-affective psychosis  Mood disorders  Conduct or non-psychotic disorders | N/A | Thematic analysis  Focus groups | 3 | N |
| Esterberg, 2005 [40]  USA | Schizophrenia  Schizophreniform disorder  Schizoaffective disorder | N/A | Traditional thematic methodology guided by transtheoretical model  Interviews | 3 | N |
| Rogers, 1998 [41]  UK | Schizophrenia/schizoaffective disorder | N/A | NR  Interviews | 3 | Y |
| Klein, 2019 [42]  Australia | Schizophrenia  Borderline personality disorder  BD | N/A | Thematic analysis  Interviews | 2 | N |
| Talley, 2019 [43]  USA | Schizophrenia  Schizoaffective disorder  BD  Major depressive disorder | N/A | Thematic analysis  Interviews | 2 | N |
| Van Den Heuvel, 2018 [44]  The Netherlands | BD type 1 and 2 | N/A | Modified phenomenological analysis  Interviews | 2 | N |
| Bassilios, 2014 [45]  Australia | Schizophrenia  Schizoaffective disorder | N/A | Cross-sectional interviews/template analysis  Interviews | 2 | N |
| Proudfoot, 2009 [46]  Australia | BD | N/A | Phenomenology/conceptual framework  Email conversations between study participants and ‘informed supports’ with lived experience of BD | 2 | N |
| Roe, 2009 [47]  Israel | Schizophrenia spectrum disorder  BD | N/A | Thematic content analysis  Interviews | 2 | N |
| Wang, 2009 [48]  New Zealand | BD type 1 and 2 | N/A | Analysis guided by inductive approach  Interviews | 2 | N |
| Carless, 2008a [49]  UIK | SMI not specified | N/A | Ethnography/categorical content analysis  Interviews  Focus groups | 2 | N |
| Jonsson, 2008 [50]  Sweden | BD type 1 and 2 | N/A | Qualitative content analysis  Interviews | 2 | N |
| Carrick, 2004 [51]  UK | Schizophrenia  Schizoaffective disorder  Psychotic disorder  Borderline personality disorder | N/A | Grounded theory  Interviews  Focus groups | 2 | N |
| Morton, 2018 [52]  Canada | BD | N/A | Thematic analysis  Interviews | 1 | N |
| Wilson, 2018 [53]  New Zealand | BD | N/A | Discourse analysis using Foucault’s framework  Interviews | 1 | N |
| Berry, 2017 [54]  UK | SMI not specified (focus groups conducted with HCPs) | N/A | Thematic analysis  Focus groups | 1 | N |
| Matthews, 2017 [55]  USA | DB type 1 and 2  DB not otherwise specified | N/A | Thematic analysis  Interviews | 1 | N |
| Radohl, 2016 [56]  USA | Schizophrenia  Schizoaffective disorder  Mood disorder with psychotic features  Disorders that cause psychosis or severe disability | N/A | Naturalistic enquiry approach/analysis using a feminisms lens and constant comparative method  Interviews | 1 | N |
| Blanner, 2015 [57]  Denmark | Schizophrenia  Substance use disorder comorbid to a psychiatric disorder | N/A | Template approach  Focus groups | 1 | N |
| Carson, 2015 [58]  USA | Schizophrenia  BD  Depression | N/A | Grounded theory  Interviews | 1 | N |
| Liersch-Sumskis, 2015 [59]  Australia | Schizophrenia | N/A | Psychophenomenology  Interviews | 1 | N |
| Morrison, 2015 [60]  Australia | Schizophrenia  BD | N/A | Phenomenology  Interviews | 1 | N |
| McKibbin, 2014 [61]  USA | SMI not specified (focus groups conducted with HCPs) | N/A | Grounded theory  Focus groups | 1 | N |
| Cabassa, 2013 [62]  USA | Schizophrenia  BD  Depressive disorder | N/A | Photovoice approach/analysis using pile sorting and constant comparative methods (grounded theory)  Photovoice groups | 1 | N |
| Leutwyler, 2013b [63]    USA | SMI not specified (interviews conducted with HCPs) | N/A | Grounded theory/constant comparison analysis approach  Interviews | 1 | N |
| Todd, 2013 [64]  UK | BD type 1 and 2 | N/A | Thematic analysis  Focus groups | 1 | N |
| Wang, 2012 [65]  New Zealand | BD type 1 and 2 | N/A | Analysis guided by content and thematic approaches  Interviews | 1 | N |
| Salyers, 2009 [66]  USA | Schizophrenia  Schizoaffective disorder  BD  Major depression  Other | N/A | Thematic analysis  Audio recordings of psychiatric visits | 1 | N |
| Sajatovic, 2005 [67]  USA | BD | N/A | Thematic analysis  Focus groups | 1 | N |
| Alverson, 1995 [68]  USA | SMI not specified | N/A | Ethnography  Interviews | 1 | N |

Abbreviations: BD – bipolar disorder; COPD – chronic obstructive pulmonary disorder; DM – diabetes mellitus (‘diabetes’); DRS - Data Richness Score (score 1-5 based on Ames et al 2017 scale); HCP – health care professional; LTC – long-term condition (physical); MH – mental health; N/A – not applicable; NR – not reported; PTSD – post-traumatic stress disorder; SMI – severe mental illness, T1DM – type 1 diabetes mellitus; T2DM – type 2 diabetes mellitus

1. Cimo A, Dewa CS. symptoms of mental illness and their impact on managing type 2 diabetes in adults. Canadian Journal of Diabetes. 2018;42(4):372-81. doi: 10.1016/j.jcjd.2017.08.256. PubMed PMID: 130791478. Language: English. Entry Date: 20180724. Revision Date: 20180724. Publication Type: Article. Journal Subset: Biomedical.

2. Knyahnytska Y, Williams C, Dale C, Webster F. changing the conversation: diabetes management in adults with severe mental illnesses and type 2 diabetes. Can. 2018. doi: <https://dx.doi.org/10.1016/j.jcjd.2018.02.001>.

3. Mulligan K, McBain H, Lamontagne-Godwin F, Chapman J, Haddad M, Jones J, et al. Barriers and enablers of type 2 diabetes self-management in people with severe mental illness. Health Expect. 2017;20(5):1020-30. Epub 2017/03/18. doi: 10.1111/hex.12543. PubMed PMID: 28306182; PubMed Central PMCID: PMCPMC5600230.

4. Blixen CE, Kanuch S, Perzynski AT, Thomas C, Dawson NV, Sajatovic M. Barriers to Self-management of Serious Mental Illness and Diabetes. Am J Health Behav. 2016;40(2):194-204. Epub 2016/03/05. doi: 10.5993/AJHB.40.2.4. PubMed PMID: 26931751; PubMed Central PMCID: PMCPMC4928189.

5. El-Mallakh P. evolving self-care in individuals with schizophrenia and diabetes mellitus. Arch Psychiatr Nurs. 2006;20(2):55-64.

6. Stenov V, Joensen LE, Knudsen L, Lindqvist Hansen D, Willaing Tapager I. "Mental Health Professionals Have Never Mentioned My Diabetes, They Don't Get Into That": A Qualitative Study of Support Needs in Adults With Type 1 and Type 2 Diabetes and Severe Mental Illness. Can. 2020;44(6):494-500. doi: <https://dx.doi.org/10.1016/j.jcjd.2020.02.006>.

7. Lowndes R, Angus J, Peter E. diabetes care and mental illness: constraining elements to physical activity and social participation in a residential care facility. Can. 2013;37(4):220-5. doi: <https://dx.doi.org/10.1016/j.jcjd.2013.03.361>.

8. Arbour-Nicitopoulos KP, Faulkner GE, Cohn TA, Selby P. smoking cessation in women with severe mental illness: exploring the role of exercise as an adjunct treatment. Arch Psychiatr Nurs. 2011;25(1):43-52. doi: <https://dx.doi.org/10.1016/j.apnu.2010.05.006>.

9. Jimenez DE, Thomas L, Bartels SJ. the role of serious mental illness in motivation, participation and adoption of health behavior change among obese/sedentary latino adults. Ethn Health. 2017:1-8. doi: <https://dx.doi.org/10.1080/13557858.2017.1390552>.

10. Chee G-L, Wynaden D, Heslop K. The physical health of young people experiencing first-episode psychosis: Mental health consumers' experiences. International Journal of Mental Health Nursing. 2019;28(1):330-8. doi: <http://dx.doi.org/10.1111/inm.12538>.

11. Nakanishi M, Tanaka S, Kurokawa G, Ando S, Yamasaki S, Fukuda M, et al. Inhibited autonomy for promoting physical health: Qualitative analysis of narratives from persons living with severe mental illness. BJPsych Open Vol 5 2019, ArtID e10. 2019;5(1):e10. doi: <http://dx.doi.org/10.1192/bjo.2018.77>.

12. Blixen C, Sajatovic M, Moore DJ, Depp C, Cushman C, Cage J, et al. Patient Participation in the Development of a Customized M-Health Intervention to Improve Medication Adherence in Poorly Adherent Individuals with Bipolar Disorder (BD) and Hypertension (HTN). Int J Healthc. 2018;4(1):25-35. doi: 10.5430/ijh.v4n1p25. PubMed PMID: 30410985.

13. Wheeler AA-OX, Roennfeldt H, Slattery M, Krinks R, Stewart V. Codesigned recommendations for increasing engagement in structured physical activity for people with serious mental health problems in Australia. (1365-2524 (Electronic)).

14. Blixen C, Perzynski AT, Bukach A, Howland M, Sajatovic M. patients' perceptions of barriers to self-managing bipolar disorder: a qualitative study. International Journal of Social Psychiatry. 2016;62(7):635-44. doi: <http://dx.doi.org/10.1177/0020764016666572>. PubMed PMID: 2016-52031-006.

15. Rastad C, Martin C, Asenlof P. barriers, benefits, and strategies for physical activity in patients with schizophrenia. Phys Ther. 2014;94(10):1467-79. doi: <https://dx.doi.org/10.2522/ptj.20120443>.

16. Shor R, Shalev A. identifying barriers to improving the wellness of persons with severe mental illness in community residential mental health facilities. Social Work in Mental Health. 2013;11(4):334-48. doi: <http://dx.doi.org/10.1080/15332985.2013.779360>. PubMed PMID: 2013-19323-002.

17. Johnstone R, Nicol K, Donaghy M, Lawrie S. barriers to uptake of physical activity in community-based patients with schizophrenia. Journal of Mental Health. 2009;18(6):523-32. doi: 10.3109/09638230903111114. PubMed PMID: 105268493. Language: English. Entry Date: 20100122. Revision Date: 20150711. Publication Type: Journal Article.

18. Keller-Hamilton BA-O, Moe AM, Breitborde NJK, Lee A, Ferketich AK. Reasons for smoking and barriers to cessation among adults with serious mental illness: A qualitative study. (1520-6629 (Electronic)).

19. Ehrlich C, Chester P, Kisely S, Crompton D, Kendall E. making sense of self-care practices at the intersection of severe mental illness and physical health-an australian study. Health & social care in the community. 2018;26(1):e47-e55. doi: 10.1111/hsc.12473. PubMed PMID: 126819758. Language: English. Entry Date: 20171219. Revision Date: 20180615. Publication Type: Article.

20. Heffner JL, Watson NL, McClure JB, Anthenelli RM, Hohl S, Bricker JB. "i smoke like this to suppress these issues that are flaws of my character": challenges and facilitators of cessation among smokers with bipolar disorder. Journal of dual diagnosis. 2018;14(1):32-9. doi: <http://dx.doi.org/10.1080/15504263.2017.1390278>. PubMed PMID: 2018-24590-004.

21. Lundstrom S, Ahlstrom BH, Jormfeldt H, Eriksson H, Skarsater I. the meaning of the lived experience of lifestyle changes for people with severe mental illness. Issues in mental health nursing. 2017;38(9):717-25. doi: <https://dx.doi.org/10.1080/01612840.2017.1330909>.

22. Firth J, Carney R, Jerome L, Elliott R, French P, Yung AR. the effects and determinants of exercise participation in first-episode psychosis: a qualitative study. BMC Psychiatry. 2016;16:36. doi: <https://dx.doi.org/10.1186/s12888-016-0751-7>.

23. Sundgren E, Hallqvist J, Fredriksson L. health for smokers with schizophrenia - a struggle to maintain a dignified life. Disabil Rehabil. 2016;38(5):416-22. doi: <https://dx.doi.org/10.3109/09638288.2015.1044033>.

24. Kemp V, Fisher C, Lawn S, Battersby M, Isaac MK. small steps: physical health promotion for people living with mental illness. International Journal of Mental Health Promotion. 2015;17(2):97-112. doi: <http://dx.doi.org/10.1080/14623730.2015.1010370>. PubMed PMID: 2015-14930-004.

25. Van den Heuvel SC, Goossens PJ, Terlouw C, Van Achterberg T, Schoonhoven L. identifying and describing patients' learning experiences towards self-management of bipolar disorders: a phenomenological study. Journal of psychiatric and mental health nursing. 2015;22(10):801-10. doi: <https://dx.doi.org/10.1111/jpm.12243>.

26. Villaggi B, Provencher H, Coulombe S, Meunier S, Radziszewski S, Hudon C, et al. Self-Management Strategies in Recovery From Mood and Anxiety Disorders. Global Qualitative Nursing Research. 2015;2:2333393615606092. doi: 10.1177/2333393615606092.

27. Leutwyler H, Hubbard EM, Slater M, Jeste DV. "it's good for me": physical activity in older adults with schizophrenia. Community Ment Health J. 2014;50(1):75-80. doi: <https://dx.doi.org/10.1007/s10597-013-9613-7>.

28. Pearsall R, Hughes S, Geddes J, Pelosi A. Understanding the problems developing a healthy living programme in patients with serious mental illness: a qualitative study. BMC Psychiatry. 2014;14:38. Epub 2014/02/15. doi: 10.1186/1471-244x-14-38. PubMed PMID: 24524248; PubMed Central PMCID: PMCPmc4098648.

29. Rönngren YM, Björk A Fau - Haage D, Haage D Fau - Kristiansen L, Kristiansen L. LIFEHOPE.EU: lifestyle and healthy outcome in physical education. (1365-2850 (Electronic)).

30. Katakura N, Matsuzawa K, Ishizawa K, Takayanagi C. psychological and physical self-management of people with schizophrenia in community psychiatric rehabilitation settings: a qualitative study. Int J Nurs Pract. 2013;19 Suppl 2:24-33. doi: <https://dx.doi.org/10.1111/ijn.12041>.

31. Leutwyler H, Hubbard EM, Jeste DV, Vinogradov S. "we're not just sitting on the periphery": a staff perspective of physical activity in older adults with schizophrenia. Gerontologist. 2013;53(3):474-83. doi: <https://dx.doi.org/10.1093/geront/gns092>.

32. Wardig RE, Bachrach-Lindstrom M, Foldemo A, Lindstrom T, Hultsjo S. prerequisites for a healthy lifestyle-experiences of persons with psychosis. Issues in mental health nursing. 2013;34(8):602-10. doi: <https://dx.doi.org/10.3109/01612840.2013.790525>.

33. Williams P, Lloyd C, King R, Paterson M. street soccer programme participation: experiences of young people with psychosis. International Journal of Therapy & Rehabilitation. 2013;20(12):606-11. PubMed PMID: 104169707. Language: English. Entry Date: 20131209. Revision Date: 20150820. Publication Type: Journal Article.

34. Barre LK, Ferron JC, Davis KE, Whitley R. Healthy eating in persons with serious mental illnesses: understanding and barriers. Psychiatr Rehabil J. 2011;34(4):304-10. Epub 2011/04/05. doi: 10.2975/34.4.2011.304.310. PubMed PMID: 21459746.

35. Suto M, Murray G, Hale S, Amari E, Michalak EE. what works for people with bipolar disorder? tips from the experts. J Affect Disord. 2010;124(1-2):76-84. doi: <https://dx.doi.org/10.1016/j.jad.2009.11.004>.

36. Schmutte T, Flanagan E Fau - Bedregal L, Bedregal L Fau - Ridgway P, Ridgway P Fau - Sells D, Sells D Fau - Styron T, Styron T Fau - Davidson L, et al. Self-efficacy and self-care: missing ingredients in health and healthcare among adults with serious mental illnesses. (0033-2720 (Print)).

37. Carless D. phases in physical activity initiation and maintenance among men with serious mental illness. International Journal of Mental Health Promotion. 2007;9(2):17-27. doi: <http://dx.doi.org/10.1080/14623730.2007.9721835>. PubMed PMID: 2007-09238-003.

38. Soundy A, Faulkner G, Taylor A. exploring variability and perceptions of lifestyle physical activity among individuals with severe and enduring mental health problems: a qualitative study. Journal of Mental Health. 2007;16(4):493-503. doi: <http://dx.doi.org/10.1080/09638230701482345>. PubMed PMID: 2007-12119-006.

39. McDevitt J, Snyder M Fau - Miller A, Miller A Fau - Wilbur J, Wilbur J. Perceptions of barriers and benefits to physical activity among outpatients in psychiatric rehabilitation. 2006;(1527-6546 (Print)).

40. Esterberg M. smoking behaviour in persons with a schizophrenia-spectrum disorder: a qualitative investigation of the transtheoretical model. 2005.

41. Rogers A, Day JC, Williams B, Randall F, Wood P, Healy D, et al. the meaning and management of neuroleptic medication: a study of patients with a diagnosis of schizophrenia. Soc Sci Med. 1998;47(9):1313-23.

42. Klein P, Lawn S, Tsourtos G, van Agteren J. Tailoring of a Smartphone Smoking Cessation App (Kick.it) for Serious Mental Illness Populations: Qualitative Study. JMIR Hum Factors. 2019;6(3):e14023-e. doi: 10.2196/14023. PubMed PMID: 31482850.

43. Talley RM, Rolin SA, Trejo BN, Goldman ML, Alves-Bradford JE, Dixon LB. Perspectives of Individuals With Serious Mental Illness on a Reverse-Colocated Care Model: A Qualitative Study. 2019;(1557-9700 (Electronic)).

44. van den Heuvel S, Goossens P, Terlouw CA-O, Schoonhoven L, van Achterberg T. Informal Caregivers' Learning Experiences With Self-Management Support of Individuals Living With Bipolar Disorder: A Phenomenological Study. 2018;(1532-5725 (Electronic)).

45. Bassilios B, Judd F, Pattison P. why don't people diagnosed with schizophrenia spectrum disorders (ssds) get enough exercise? Australas. 2014;22(1):71-7. doi: <https://dx.doi.org/10.1177/1039856213510575>.

46. Proudfoot JG, Parker GB, Benoit M, Manicavasagar V, Smith M, Gayed A. what happens after diagnosis? understanding the experiences of patients with newly-diagnosed bipolar disorder. Health expectations : an international journal of public participation in health care and health policy. 2009;12(2):120-9. doi: <https://dx.doi.org/10.1111/j.1369-7625.2009.00541.x>.

47. Roe D, Goldblatt H, Baloush-Klienman V, Swarbrick M, Davidson L. why and how people decide to stop taking prescribed psychiatric medication: exploring the subjective process of choice. Psychiatric rehabilitation journal. 2009;33(1):38-46. doi: <https://dx.doi.org/10.2975/33.1.2009.38.46>.

48. Wang G, Tse S, Michalak EE, Depp C. self-management techniques for bipolar disorder in a sample of new zealand chinese...including commentary by depp c. International Journal of Therapy & Rehabilitation. 2009;16(11):602-8. PubMed PMID: 105236446. Language: English. Entry Date: 20100101. Revision Date: 20150820. Publication Type: Journal Article.

49. Carless D, Douglas K. the contribution of exercise and sport to mental health promotion in serious mental illness: an interpretive project. International Journal of Mental Health Promotion. 2008;10(4):5-12. doi: <http://dx.doi.org/10.1080/14623730.2008.9721771>. PubMed PMID: 2008-17491-002.

50. Jonsson PD, Wijk H, Skarsater I, Danielson E. persons living with bipolar disorder--their view of the illness and the future. Issues in mental health nursing. 2008;29(11):1217-36. doi: <https://dx.doi.org/10.1080/01612840802370764>.

51. Carrick R, Mitchell A, Powell RA, Lloyd K. the quest for well-being: a qualitative study of the experience of taking antipsychotic medication. Psychol Psychother. 2004;77(Pt 1):19-33.

52. Morton E, Michalak EE, Hole R, Buzwell S, Murray G. 'taking back the reins'-a qualitative study of the meaning and experience of self-management in bipolar disorder. Journal of Affective Disorders. 2018;228:160-5. doi: <http://dx.doi.org/10.1016/j.jad.2017.12.018>. PubMed PMID: 2018-05040-022.

53. Wilson L, Crowe M, Scott A, Lacey C. self‐management for bipolar disorder and the construction of the ethical self. Nursing Inquiry. 2018;25(3):1-. doi: 10.1111/nin.12232. PubMed PMID: 130696408. Language: English. Entry Date: 20180716. Revision Date: 20180719. Publication Type: Article. Journal Subset: Australia & New Zealand.

54. Berry N, Bucci S, Lobban F. use of the internet and mobile phones for self-management of severe mental health problems: qualitative study of staff views. JMIR Ment Health. 2017;4(4):e52. doi: <https://dx.doi.org/10.2196/mental.8311>.

55. Matthews M, Murnane E, Snyder J. quantifying the changeable self: the role of self-tracking in coming to terms with and managing bipolar disorder. Human-Computer Interaction. 2017;32(5-6):413-46. doi: <http://dx.doi.org/10.1080/07370024.2017.1294983>. PubMed PMID: 2017-43368-008.

56. Radohl T. living a meaningful life with serious mental illness: defining and understanding personal medicine. Journal of the Society for Social Work and Research. 2016;7(2):345-69. doi: <http://dx.doi.org/10.1086/686807>. PubMed PMID: 2016-26008-010.

57. Blanner Kristiansen C, Juel A, Vinther Hansen M, Hansen AM, Kilian R, Hjorth P. promoting physical health in severe mental illness: patient and staff perspective. Acta psychiatrica Scandinavica. 2015;132(6):470-8. doi: <https://dx.doi.org/10.1111/acps.12520>.

58. Carson NE, Blake CE, Saunders R. perceptions and dietary intake of self-described healthy and unhealthy eaters with severe mental illness. Community Mental Health Journal. 2015;51(3):281-8. doi: <http://dx.doi.org/10.1007/s10597-014-9806-8>. PubMed PMID: 2014-57335-001.

59. Liersch-Sumskis S, Moxham L, Curtis J. choosing to use compared to taking medication: the meaning of medication as described by people who experience schizophrenia. Perspect Psychiatr Care. 2015;51(2):114-20. doi: <https://dx.doi.org/10.1111/ppc.12072>.

60. Morrison P, Meehan T, Stomski NJ. living with antipsychotic medication side-effects: the experience of australian mental health consumers. Int J Ment Health Nurs. 2015;24(3):253-61. doi: <https://dx.doi.org/10.1111/inm.12110>.

61. McKibbin CL, Kitchen KA, Wykes TL, Lee AA. barriers and facilitators of a healthy lifestyle among persons with serious and persistent mental illness: perspectives of community mental health providers. Community Mental Health Journal. 2014;50(5):566-76. doi: <http://dx.doi.org/10.1007/s10597-013-9650-2>. PubMed PMID: 2013-36659-001.

62. Cabassa LJ, Parcesepe A, Nicasio A, Baxter E, Tsemberis S, Lewis-Fernandez R. health and wellness photovoice project: engaging consumers with serious mental illness in health care interventions. Qualitative health research. 2013;23(5):618-30. doi: <http://dx.doi.org/10.1177/1049732312470872>. PubMed PMID: 2013-12183-004.

63. Leutwyler HC, Fox PJ, Wallhagen M. medication adherence among older adults with schizophrenia. J Gerontol Nurs. 2013;39(2):26-34; quiz 5. doi: <https://dx.doi.org/10.3928/00989134-20130109-02>.

64. Todd NJ, Jones SH, Lobban FA. what do service users with bipolar disorder want from a web-based self-management intervention? a qualitative focus group study. Clin. 2013;20(6):531-43. doi: <https://dx.doi.org/10.1002/cpp.1804>.

65. Wang GY, Henning M. family involvement in chinese immigrants with bipolar disorder in new zealand. The New Zealand medical journal. 2012;126(1368):45-52.

66. Salyers MP, Matthias MS, Spann CL, Lydick JM, Rollins AL, Frankel RM. the role of patient activation in psychiatric visits. Psychiatr Serv. 2009;60(11):1535-9. doi: <https://dx.doi.org/10.1176/appi.ps.60.11.1535>.

67. Sajatovic M, Davies M, Bauer MS, McBride L, Hays RW, Safavi R, et al. attitudes regarding the collaborative practice model and treatment adherence among individuals with bipolar disorder. Compr Psychiatry. 2005;46(4):272-7.

68. Alverson M, Becker DR, Drake RE. an ethnographic study of coping strategies used by people with severe mental illness participating in supported employment. Psychosocial Rehabilitation Journal. 1995;18(4):115-28. PubMed PMID: 107430889. Language: English. Entry Date: 19951201. Revision Date: 20150820. Publication Type: Journal Article.
